# Pre-treatment of blood samples reveal normal blood hypocretin/orexin signal in narcolepsy type 1

**DOI:** 10.1093/braincomms/fcab050

**Published:** 2021-03-22

**Authors:** Helene M Ægidius, Lars Kruse, Gitte L Christensen, Marc P Lorentzen, Niklas R Jørgensen, Monica Moresco, Fabio Pizza, Giuseppe Plazzi, Poul J Jennum, Birgitte R Kornum

**Affiliations:** 1 Department of Neuroscience, University of Copenhagen, 2200 Copenhagen N, Denmark; 2 Department of Clinical Biochemistry, Rigshospitalet, 2600 Glostrup, Denmark; 3 Istituto delle Scienze Neurologiche, Ospedale Bellaria, IRCCS Bologna, 40139 Bologna, Italy; 4 Department of Biomedical and Neuromotor Sciences (DIBINEM), University of Bologna, 40126 Bologna, Italy; 5 Department of Biomedical, Metabolic and Neural Sciences, University of Modena and Reggio-Emilia, 41121 Modena, Italy; 6 Department of Clinical Neurophysiology, Danish Center for Sleep Medicine, Rigshospitalet, 2600 Glostrup, Denmark

**Keywords:** hypocretin, orexin, plasma hypocretin-1, narcolepsy type 1, radioimmunoassay

## Abstract

The hypocretin/orexin system regulates arousal through central nervous system mechanisms and plays an important role in sleep, wakefulness and energy homeostasis. It is unclear whether hypocretin peptides are also present in blood due to difficulties in measuring reliable and reproducible levels of the peptides in blood samples. Lack of hypocretin signalling causes the sleep disorder narcolepsy type 1, and low concentration of cerebrospinal fluid hypocretin-1/orexin-A peptide is a hallmark of the disease. This measurement has high diagnostic value, but performing a lumbar puncture is not without discomfort and possible complications for the patient. A blood-based test to assess hypocretin-1 deficiency would therefore be of obvious benefit. We here demonstrate that heating plasma or serum samples to 65°C for 30 min at pH 8 significantly increases hypocretin-1 immunoreactivity enabling stable and reproducible measurement of hypocretin-1 in blood samples. Specificity of the signal was verified by high-performance liquid chromatography and by measuring blood samples from mice lacking hypocretin. Unspecific background signal in the assay was high. Using our method, we show that hypocretin-1 immunoreactivity in blood samples from narcolepsy type 1 patients does not differ from the levels detected in control samples. The data presented here suggest that hypocretin-1 is present in the blood stream in the low picograms per millilitres range and that peripheral hypocretin-1 concentrations are unchanged in narcolepsy type 1.

## Teaser

The hypocretin/orexin system in the brain regulates sleep and wakefulness. Aegidius et al. have developed a simple test to measure hypocretin-1 peptide in blood samples, and show that peripheral hypocretin-1 concentrations are unchanged in narcolepsy type 1 despite being absent in the brains of these patients.

## Graphical Abstract

**Graphical Abstract:**
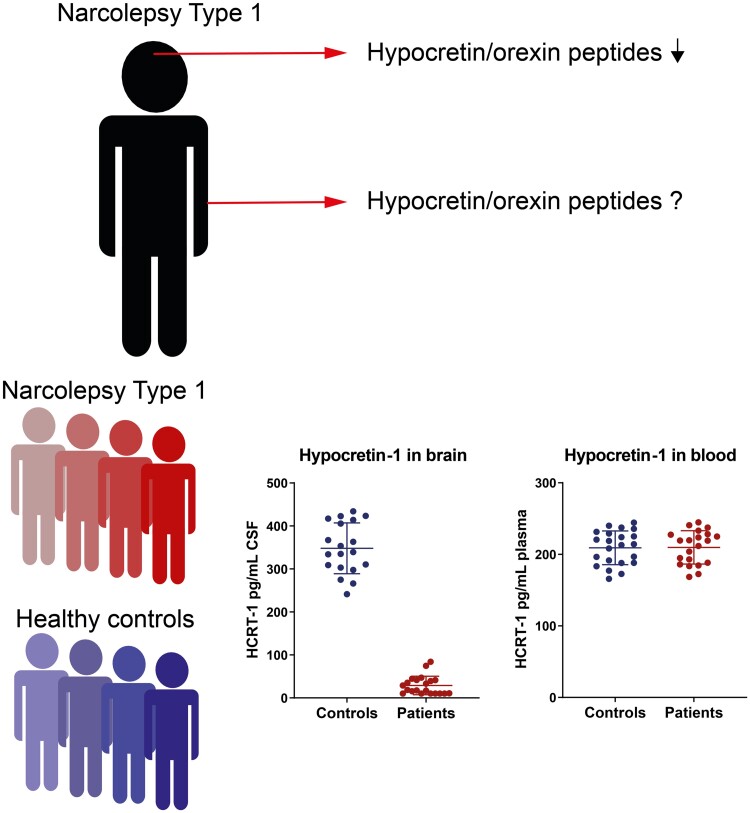

## Introduction

Since the discovery of the hypocretin/orexin (HCRT) peptides in 1998,^1^^,^^2^ much have been learned about their functions. They are implicated in a wide range of neuronal circuits, playing important roles in sleep and wake regulation, autonomic regulation, neuroendocrine and locomotor function, regulation of hormonal secretions, feeding behaviour and energy homeostasis as well as in the pathology of Narcolepsy.^3–5^ The two hypocretin neuropeptides are derived from a common precursor protein (prepro-HCRT) which is proteolytically cleaved to form the 33-amino acid peptide hypocretin-1 (HCRT-1) (3.56 kDa) and the 28-amino acid peptide hypocretin-2 (HCRT-2) (2.94 kDa).^2^ The peptides are produced by 60 000–70 000 neurons (in humans) located in the lateral hypothalamus,^6^ from where the neurons project to most areas of the brain. Interestingly, a growing body of evidence points towards a direct regulatory effect of HCRT peptides, not only in the brain, but also in peripheral tissues. HCRT receptors have been found in a variety of tissues including kidney, adipose tissue, gastrointestinal tract, pancreas, adrenal gland and reproductive organs across different species.^7–14^ The abundance and wide distribution of HCRT receptors in peripheral tissues suggests biological relevance, but for many tissues the function remains elusive. The source of peripheral HCRT is also unknown. Detection of prepro-HCRT mRNA and HCRT peptides in peripheral organs (stomach, ileum, colon, adrenal glands and pancreas) has been reported but the specificity of the signals have never been verified.^7^^,^^9^^,^^13^^,^^15^ Common for these findings is that the presence of peptide has only been shown using antibody-based technologies, and the specificity of these antibodies have been questioned.^16^ Peripheral HCRT might also derive from the central nervous system and function as a peptide hormone. It has indeed been demonstrated that HCRT-1 is highly lipophilic and can cross the blood brain barrier from blood^17^ and that injections of HCRT-1 into the CSF of the cisterna magna can reach the blood.^18^ The presence and possible transport of HCRT peptides in the blood stream has, however, been a topic of much debate. The controversy originates from difficulties in measuring reliable and reproducible levels of HCRT-1 or HCRT-2 in serum or plasma and lack of consistency and specificity of the methods/antibodies used.^19^^,^^20^Supplementary Table 1 summarizes findings from published studies claiming to measure HCRT-1 in blood (list not exhaustive). Despite applying similar methodology for detection and quantification of HCRT-1, markedly different HCRT-1 levels have been reported in both plasma and serum. To date no study of peripheral HCRT-1 levels has convincingly demonstrated that the signal observed was a genuine HCRT-1 signal.

Adding to the controversy are two conflicting papers describing measurements of plasma HCRT-1 levels in narcolepsy type 1 (NT1) patients. The sleep disorder NT1 is associated with a loss of HCRT production in the brain: low levels of HCRT-1 in the CSF is a highly specific and sensitive diagnostic marker for NT1.^21^ To test if plasma HCRT-1 could be used as a biomarker, early attempts were made towards measuring HCRT-1 in blood samples from these patients and controls. While Dalal et al.^22^ showed that plasma HCRT-1 levels were normal in narcoleptic patients Higuchi et al.^23^ showed decreased plasma levels in narcolepsy. A later commentary questioned the specificity of the assays used in the two studies.^24^ A reproducible and reliable assay that gives an authentic HCRT-1 signal from blood samples is still to be developed. Therefore, we aimed to develop and validate a simple method that allows for detection of HCRT-1 in blood. In addition, we evaluated if our assay would be useful as a diagnostic tool in NT1.

## Materials and methods

### Outline of experiments

To meet our goals, we performed the following series of experiments: (i) Assay development, where we tested a whole range of sample pre-treatments to find the optimal conditions for HCRT-1 detection. (ii) Assay validation, where we verified the specificity of the HCRT-1 signal by using different antibodies, different methodologies and finally by using blood from animals lacking HCRT-1 in blood. (iii) HCRT-1 measurements in blood from two independent cohorts of NT1 patients and clinical controls.

### Antibodies and proteins/peptides

Pierce antibody (#PA124892, Thermo Scientific—discontinued), Phoenix (#RK-003–30, Phoenix Pharmaceuticals, CA, USA), Peninsula (#T-4072.0500, Peninsula Laboratories International).

Pierce antibody immunogen: N-terminal residues 1–17 of human HCRT-1, with a disulphide bridge between cysteines 6 and 12. Phoenix antibody: N-terminal region. Peninsula antibody: N terminal region and the 16–33 region of HCRT-1. Phoenix HCRT-1 standard peptide (#RK-003–30, Phoenix Pharmaceuticals) and modified HCRT-1 peptide (Shafer-N). rHSA, recombinant human serum albumin (Sigma -#A9731, Novozymes—Recombumin). BSA, bovine serum albumin (New England Biolabs, #B9001S).

### Human samples for assay development

Blood was collected by the Department of Clinical Biochemistry at Rigshospitalet, Glostrup. All human samples were completely anonymized by pooling and by removing any personal references from the materials.

Blood samples were drawn into ethylenediaminetetraacetic acid (EDTA) or serum Vacutainer tubes (BD, Franklin, NJ, USA). EDTA samples were gently mixed and then immediately centrifuged at 2000 g for 15 min at 4°C. Serum samples were gently mixed, allowed to clot for 30 min and then centrifuged at 1500 g for 10 min at 4°C. Following clinical analysis, an estimate of 50 plasma or serum samples were pooled to obtain a plasma and serum pool, respectively. The time from collection to pick-up was 3–4 h and meanwhile the samples were kept at room temperature. After pooling, plasma and serum were aliquoted and stored at −80°C until use. For evaluation of assay interference by plasma anticoagulants and serum, blood from two individuals was drawn into citrate-, EDTA-, heparin- and serum Vacutainer tubes (BD). Plasma and serum were prepared as described above. Half of each sample were frozen for 30 min at −80°C prior to standard operating procedure (SOP) treatment, whereas the other half were SOP treated without freezing.

### Animals

C57BL/6 male and female *Hcrt*-KO (hetero- and homozygous, *n* = 10 and *n* = 26), *Hcrt-Atxn3* (*n* = 10) and wild-type (WT, *n* = 30) mice were used for these experiments. The *Hcrt-Atxn3* mice model is a HCRT-specific neurodegeneration model.^25^ Blood from these animals was drawn from the retro-orbital sinus under isoflurane anaesthesia as a terminal procedure. Blood samples were drawn into vacutainer tubes (BD) containing EDTA. Blood from two animals of the same genotype were drawn into the same tubes. Samples were gently mixed and then immediately centrifuged at 2000 g for 15 min at 4°C. Plasma was stored at −80°C until used.

### Clinical cohort from Danish Center for sleep medicine

Approval for the use of blood samples was granted by the Danish Ethical Committee (KA03119). Thirty-seven subjects were included in this study. Lumbar punctures and blood sampling were performed for diagnostic purposes after informed consent from each subject. Lumbar punctures were performed between 8 and 12 AM and blood was drawn immediately after. Blood samples were drawn into Vacutainer tubes (BD) containing EDTA. Samples were gently mixed and then immediately centrifuged at 2000 g for 15 min at 4°C. Both CSF and plasma samples were stored at −80°C. Eighteen subjects diagnosed with NT1 were included in this study and 19 non-narcoleptic controls. Controls were selected from subjects seen at the sleep clinic that did not meet the criteria for any diagnosis. A summary of subject characteristics is listed in Table 1.

**Table 1:** Cohort characteristics

	Danish cohort		Italian cohort	
	Narcolepsy type 1	Control	Narcolepsy type 1	Control
Number	18	19	21	22
Age (years)	34 ± 22	33 ± 12	23 ± 15	25 ± 17
Gender (M:F)	14:4	6:13	13:8	12:10
Body mass index (kg/m^2^)	27 ± 6.0 (*n* = 17)	25 ± 4.6	26 ± 5.7	24 ± 5.2
CSF HCRT-1 < 110 pg/ml	100%	0%	100%	0% (*n* = 19)
CSF HCRT-1 levels (pg/ml)	16.3 ± 10.3	336.6 ± 27.3	29.0 ± 21.5	348.0 ± 59.2
Albumin concentration (g/dl)	3.97 ± 0.77	3.56 ± 0.67	N/A	N/A

Characteristics of two independent cohorts from the Danish Center for Sleep Medicine and Bologna. Data reported as mean ± standard deviation. In the case of missing data, the number of individuals where data were available are given in parenthesis.

N/A = not available.

### Clinical cohort from the Center for Narcolepsy of Bologna, Italy

Approval for the use of blood samples was granted by the Comitato Etico Interaziendale Bologna-Imola (17009). The Italian cohort comprised 43 subjects, 21 subjects with an NT1 diagnosis and 22 controls. Unique to this cohort was that blood samples from narcoleptic patients were collected between 1 and 13 months from disease onset (8.4 ± 4.2 months). Controls were selected from subjects seen at the sleep clinic that did not meet the criteria for a narcolepsy type 1 or type 2 diagnosis, 17 did not meet the criteria for any diagnosis while 5 had idiopathic hypersomnia. A summary of subject characteristics is listed in Table 1.

### Assay development

Various combinations of temperature, heating time and buffer with varying pH were tested to investigate the effect of altering assay conditions on HCRT-1 signal from plasma and an HCRT-1 peptide standard. The pH of the buffers tested were pH 2 (phosphate buffer), pH 4 and 6 (acetic acid buffer), pH 8 (Tris:HCl buffer), pH 9 and 10 (sodium bicarbonate buffer). The concentration of all buffers used was 0.09 M. The heating temperature ranged from 40 to 90°C and heating time ranged from 0 to 120 min. SOP: 250 µl plasma was mixed with 250 µl Tris:HCl buffer and heated for 30 min at 65°C. HCRT-1 levels were measured by standard radioimmunoassay (RIA, #RK-003–30, Phoenix Pharmaceuticals).

### Radioimmunoassay

RIA was used for quantification of HCRT-1 levels in all plasma, serum and CSF samples during assay development and testing of the clinical cohorts. All samples were run in technical duplicates. RIA kits were purchased from Phoenix Pharmaceuticals, CA, USA (#RK-003–30). The Phoenix antibody from the kit was used for detection unless a different antibody is specified. According to the manufacturer no cross-reactivity was observed with C-terminal HCRT-1 fragment (16–33), HCRT-2, Agouti-Related Protein (83–132)-amide, Neuropeptide Y, α-MSH or Leptin. Assay quality was monitored by the internal positive control sample included in the assay. Intra-assay variability was assessed by including a control sample from a pool of human CSF in each assay. The standard range of the RIA kit is 10–1280 pg/ml. RIA standard curves are depicted as B/B0% on the *x*-axis and concentration (pg/ml) on the *y*-axis. B0 = total binding—non-specific binding. B = CPM of standard—non-specific binding. The plasma concentrations have been recalculated according to the initial plasma volume and are given in picograms per millilitres plasma. The person performing RIA was blinded to treatment conditions, group status and diagnosis.

### High-performance liquid chromatography

High-performance liquid chromatography (HPLC) was used for chromatographic characterization of HCRT-1 in plasma/serum and HCRT-1 standard peptide. Samples were loaded onto an Xselect C18 column (Waters) and eluted with a linear gradient of acetonitrile containing TFA. Fractions were collected from Minute 2 to 20 (1 ml/min) and each fraction was dried by N_2_ for 3 h and stored at −20°C until further use. Overview of the HPLC programme can be found in Supplementary Table 2.

### Western blot

Samples were heated for 10 min at 70°C in 4× LDS sample buffer or 2× Laemmli buffer, run on SDS Precast Gel 4–20% (NXG42012, Expedeon, SD, USA), and transferred to a PVDF membranes. Membranes were blocked for an hour with TBS-T buffer [150 mM NaCl, 20 mM Tris HCl (pH 7.4), 0.1% Tween 20] containing 2% ECL Prime Blocking agent (RPN2125, GE Healthcare, UK). The membranes were incubated overnight with primary antibody in TBS-T containing 2% ECL Prime Blocking agent (Phoenix and Peninsula 1:50, Pierce 1:10 000). Then the membranes were washed five times with TBS-T and incubated with horseradish peroxidase-conjugated anti-rabbit IgG diluted 1:40 000 (NA934 GE healthcare) in TBS-T containing 2% ECL Prime Blocking agent. The proteins were visualized by ECL Select (RPN2235, GE Healthcare).

### Immunoprecipitation for HPLC analysis

Immunoprecipitation (IP) of plasma samples were performed according to the RIA protocol except that addition of ^125^I-hcrt was omitted. In brief, SOP treated plasma was mixed with Phoenix antibody and incubated overnight at 4°C (22 h). After incubation Goat Anti-Rabbit IgG serum and Normal Rabbit Serum was added to the tubes. Tubes were vortexed and incubated at room temperature for 90 min. RIA buffer was added to the tubes and centrifuged at 1700 g for 20 min at 4°C. The supernatant was discarded and samples were stored at −20°C until use. Ten IP samples were dissolved in a total of 120 µl mobile phase A reagent (5% MeCN:95% H_2_O:0.1% TFA) and 99 µl of the solution was injected to the HPLC apparatus and run according to the HPLC program shown in Supplementary Table 2.

### Testing of clinical cohorts

Prior to HCRT-1 quantification by RIA, plasma samples were pre-treated according to the developed SOP. RIA was initiated directly after pre-treatment. All plasma and CSF samples were analysed in technical duplicates. To ensure least amount of variation between samples, all samples were analysed in one RIA setup. The mean CV% was 2.5% and 2.2% for CSF and plasma, respectively. The mean CV% for the Italian cohort was 6.6% for plasma. The person performing pre-treatment of plasma, RIA and data analysis was blinded to diagnosis.

### Data analysis

Results are reported as mean ± SD. HCRT-1 plasma levels in NT1 and controls were compared using Student’s *t*-test. For comparison of Hcrt-1 (mouse hypocretin-1) plasma levels of *Hcrt*-KO, *Hcrt-Atxn3* and WT mice and during assay development a one-way ANOVA with a *posthoc* multiple comparison test was used. To access relationship between HCRT-1 levels in plasma and relevant variables of the clinical cohorts, we performed a multiple regression analysis. For the Danish cohort, variables considered for the analyses were HCRT-1 plasma levels, BMI, gender, age, diagnose, plasma storage time and albumin concentration in plasma. These variables were deemed appropriate based on previous literature. For the Italian cohort, variables considered for the analysis were HCRT-1 plasma levels, BMI, gender, age and diagnose. For the sub-analysis of NT1 patients from the Italian cohort, HCRT-1 plasma and CSF levels, BMI, age, diagnose and time of blood sampling from disease onset were included. In each model, assumption of linearity, independence of errors, homoscedasticity and normality of residuals were tested. For statistical analysis, a *P*-value <0.05 was considered statistically significant (two-sided). All data analyses were done using the statistical program RStudio.^26^

### Data availability

Data are available upon reasonable request.

## Results

### Separation from protein carriers in blood aids in detection of HCRT-1

**Figure 1:**
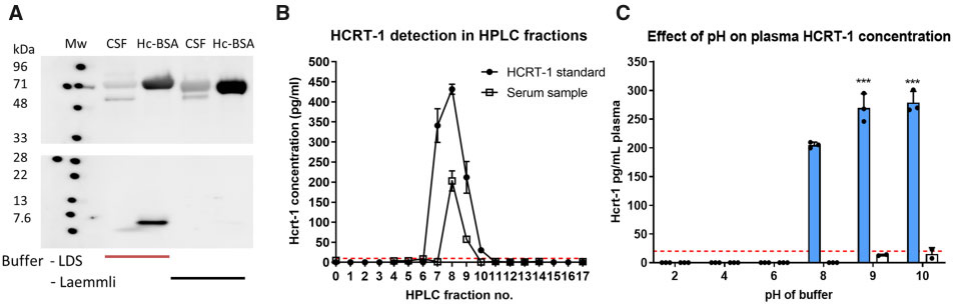
**Dissociation from protein carriers in blood aids in immunodetection of HCRT-1.** (**A**) Western blot detection of HCRT-1 and albumin. First two lanes depict treatment of pooled CSF and HCRT-1 standard (contains BSA, thus labelled Hc-BSA) with 50 mM DTT-containing LDS reducing and denaturing sample buffer. The last two lanes are the same samples treated with conventional Leammli with beta-mercaptoethanol buffer. The blot shows strong HCRT-1 signal only in the sample treated with LDS buffer, indicating that dissociation from albumin is necessary for detection. Gel has been cropped. Full gel can be found in Supplementary Fig. 2. (**B**) HPLC fractionation of HCRT-1 standard or 99 µl serum/H20 (1:1) followed by detection of HCRT-1 in individual fractions by RIA. (**C**) Neutral to basic solution conditions provides the best conditions for HCRT-1 detection. Samples were heated for 10 min at 65°C. *n* = 3 for plasma and buffer group and *n* = 2 for buffer group. ***P <0.001 compared with plasma and buffer pH = 8 group. One-way ANOVA with Dunnett’s multiple comparison test: overall effect *F*(5,12) = 352.9, *P* <0.0001. BSA = bovine serum albumin; HCRT-1 = hypocretin-1; HPLC = high-performance liquid chromatography.

We hypothesized that the presence of HCRT-1 in blood is masked in the assays used today due to an interaction between HCRT-1 and protein carriers in blood. This is a common feature of steroid and lipophilic hormones transported in plasma.^27^ Facilitating dissociation of HCRT-1 from such protein carriers should allow for detection of HCRT-1 in blood. To test this, we took advantage of the fact that the HCRT-1 standard peptide provided with the RIA assay used in the clinic, is mixed with albumin as protecting factor. Using this we performed western blot using antibodies detecting HCRT-1 and albumin on the HCRT-1/albumin mix and on CSF (Fig. 1A). Both samples were treated with two different buffers: (i) a 50 mM DTT-containing LDS reducing and denaturing sample buffer or (ii) a Leammli buffer with beta-mercaptoethanol. The blot shows strong immunoreactivity at the expected size of albumin, and HCRT-1 signal only in the sample treated with LDS buffer indicating that dissociation from albumin increases free HCRT-1 in the standard. No HCRT-1 signal was seen in the CSF sample demonstrating the low sensitivity of western blotting.

RIA detection is well known to have a much higher sensitivity compared with western blot, and can detect HCRT-1 in CSF with a detection range of 40–500 pg/ml. The standard RIA protocol does, however, not show a reproducible HCRT-1 signal from crude plasma or serum. In most runs we did not see any signal from plasma or serum, but we occasionally did get a signal and in these cases the reproducibility was low. Instead, we speculated whether HPLC fractionation of plasma or serum to separate blood constituents based on physiochemical properties would allow for detection of HCRT-1. Indeed, HPLC fractionation of a serum sample followed by detection of HCRT-1 in the individual fractions by RIA revealed a positive signal in fraction 8 and 9 corresponding to what was obtained with the HCRT-1 standard (Fig. 1B). This shows that it is possible to detect HCRT-1 in blood samples following HPLC separation. Additionally, SDS-page confirmed that HPLC fractionation facilitated separation of HCRT-1 from albumin which eluted later than what HCRT-1 did (Supplementary Fig. 1A). Similar was observed from the HPLC chromatogram (Supplementary Fig. 1B).

Taken together, our data suggest that dissociation of HCRT-1 from protein carriers allows for its detection in blood samples.

Drawbacks to HPLC fractionation were poor column lifetime and lack of reproducibility. After one HPLC run, the retaining properties of the column seemed lost. Plasma and serum samples indeed contain a significant amount of salt and proteins that can precipitate or adsorb on the packing material, eventually clogging the column. Incidence of clogging is high when crude plasma or serum is injected directly into the column as it was in these experiments. Simple sample pre-treatment, such as protein precipitation with acetic acid, prior to HPLC did not solve this problem since the HCRT-1 signal then was lost. The reason for this is likely that HCRT-1 precipitates together with its protein carriers. To avoid these problems, we explored other means of dissociating HCRT-1 from its protein carriers.

### pH-change and heat treatment of plasma increases HCRT-1 signal

**Figure 2:**
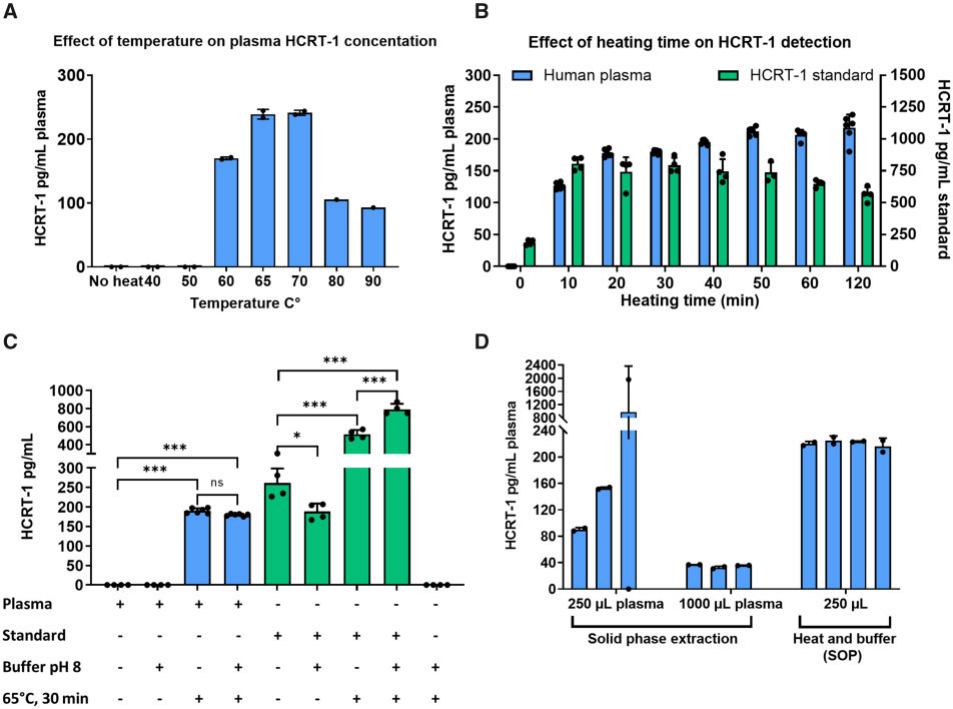
**Simple pre-treatment allows for reproducible detection of HCRT-1 in plasma.** (**A**) Effect of temperature on plasma HCRT-1 detection. Samples were heated at pH = 8, *n* = 2. (**B**) Increasing heating time increases plasma HCRT-1, *n* = 6 for plasma (left *y*-axis) and *n* = 4 for standard (right *y*-axis). Samples were heated at 65°C at pH = 8. (**C**) Combined effect of heat and/or pH treatment. Heat but not pH-buffering is necessary for detection of HCRT-1 in plasma, *n* = 6 for samples with plasma. Remaining groups *n* = 4, *P = 0.016, ***P < 0.001. One-way ANOVA with Tukey’s multiple comparison test: overall effect *F*(8,31) = 379.2, *P* < 0.0001. (**D**) SPE versus heat and pH-change (heat and buffer). The assay developed gives rise to a higher detectable HCRT-1 concentration in plasma compared with SPE. Shown are individual samples with two technical replicates. HCRT-1 was quantified by RIA in duplicates for all samples. HCRT-1 = hypocretin-1; SPE = solid phase extraction.

The strength of intermolecular interactions holding a ligand−protein complex together can be affected by a range of external factors such as pH, heat, hydrophobic surfaces, high shear or the presence of metal ions. Altering assay conditions can therefore significantly change the binding affinity of a ligand−protein complex and facilitate dissociation. We hypothesized, that a change in pH and/or heating of plasma would facilitate dissociation of HCRT-1 from protein carriers in the blood and that would in turn allow for detection of HCRT-1. We indeed found that changing the pH of the plasma solution (whilst heating the plasma sample) influenced HCRT-1 detection (Fig. 1C). At pH ≤6 no HCRT-1 signal was detectable, whereas a signal was observed in the pH range of 8–10 suggesting that basic pH provides the best conditions for HCRT-1 dissociation and detection. The highest HCRT-1 yield was obtained at pH 9 and 10, but due to increased background signal, we chose pH 8 for achieving the most reliable HCRT-1 signal. Similarly changing the pH of the HCRT-1 standard increased the HCRT-1 signal compared with untreated (−) HCRT-1 standard (Supplementary Fig. 3B). This is in agreement with the fact that the HCRT-1 standard contains albumin as carrier. Heating temperature also affected the HCRT-1 signal in plasma (Fig. 2A). Below 60°C no HCRT-1 signal was detectable. The highest HCRT-1 yield was obtained with heating temperatures of 65°C and 70°C. Temperatures exceeding 70°C was accompanied by a decrease in HCRT-1 signal compared with the 65–70°C range, likely due to general protein precipitation or denaturation. Figure 2B shows the effect of heating time on HCRT-1 yield for plasma and HCRT-1 standard. Increasing heating time resulted in an increase in HCRT-1 yield. However, exceeding 30 min decreased the HCRT-1 signal from the HCRT-1 standard. Based on these initial experiments, a buffer of pH 8 and heating for 30 min at 65°C was chosen as the SOP. Figure 2C summarizes the effect of such treatment on the detectable signal from plasma HCRT-1 and HCRT-1 standard. Our data suggest that heat and not buffer is the main contributor to HCRT-1 dissociation and detection. No signal was observed if plasma was not exposed to heat.

CSF is a much less complicated matrix compared with blood, and in CSF HCRT-1 is readily measurable with RIA with no pre-treatment. This could be due to much lower protein binding of HCRT-1 in CSF as CSF albumin concentration is <0.5 g/l while it is 35–50 g/l in plasma. To investigate this, we tested the effect of SOP treatment on CSF HCRT-1 levels of four patients from the Danish clinical cohort. CSF samples from two NT1 patient with established CSF HCRT-1 level <10 pg/ml and two controls >160 pg/ml were tested. SOP treatment of the samples increased the HCRT-1 signal, but to a very low extend (Supplementary Fig. 4). Both untreated and treated CSF of NT1 patients were below the standard range of the assay, <10 pg/ml. This has two important implications. First the increased HCRT-1 signal observed following SOP treatment of plasma cannot be attributed to an increased antigenicity of HCRT-1 per se altering antibody binding. It also shows that our treatment does not cause a false positive signal from the formation of new non-HCRT-1 epitopes as a result of protein denaturation of proteins present in CSF.

Since the SOP treatment gave rise to an increased signal from the HCRT-1 standard with albumin as carrier, we wanted to test whether albumin itself, the most abundant protein in blood, would give rise to a false positive signal in our setup. We tested untreated and SOP treated BSA and recombinant human serum albumin. For all samples, no HCRT-1 signal was observed following treatment, suggesting that the increased signal following SOP treatment of the HCRT-1 plasma or standard is not caused by the presence of albumin (data not shown).

Previous efforts to measure HCRT-1 in plasma have mainly been based on solid phase extraction (SPE) of acidified plasma loaded onto a C18 columns prior to quantification using either RIA or enzyme immunoassays. However, in our hands this approach showed varying results and low reproducibility. Figure 2D shows two SPE experiments and a SOP treatment experiment with the same plasma pool. Our assay gives rise to a higher and a more reproducible signal compared with the conventional SPE protocol. Increasing plasma volume to 1000 µl in the SPE protocol did not give rise to a higher signal than 250 µl further questioning this method due to lack of linearity.

Assay linearity in our case was tested in a linearity-of-dilution experiment, where seven different plasma dilutions were included (Supplementary Fig. 5A). A linear relationship between the observed HCRT-1 levels in plasma and the expected HCRT-1 levels was established with a correlation coefficient of 0.991, indicating that the assay has little to no matrix interference.

Assay interference by plasma anticoagulants and serum were finally evaluated to select the best blood collecting system for blood sampling as was sensitivity to freezing. Citrate-, EDTA- and heparin plasma plus serum was collected at the same time from two healthy individuals, and half of each sample was frozen for 30 min at −80°C. The samples were next SOP treated and HCRT-1 levels were quantified (Supplementary Fig. 5B). None of the anticoagulants nor serum significantly changed the detected HCRT-1 levels indicating that assay interference by the type of blood collection system is low for this assay.

### Validation of specificity of HCRT-1 immunodetection after sample pre-treatment

**Figure 3:**
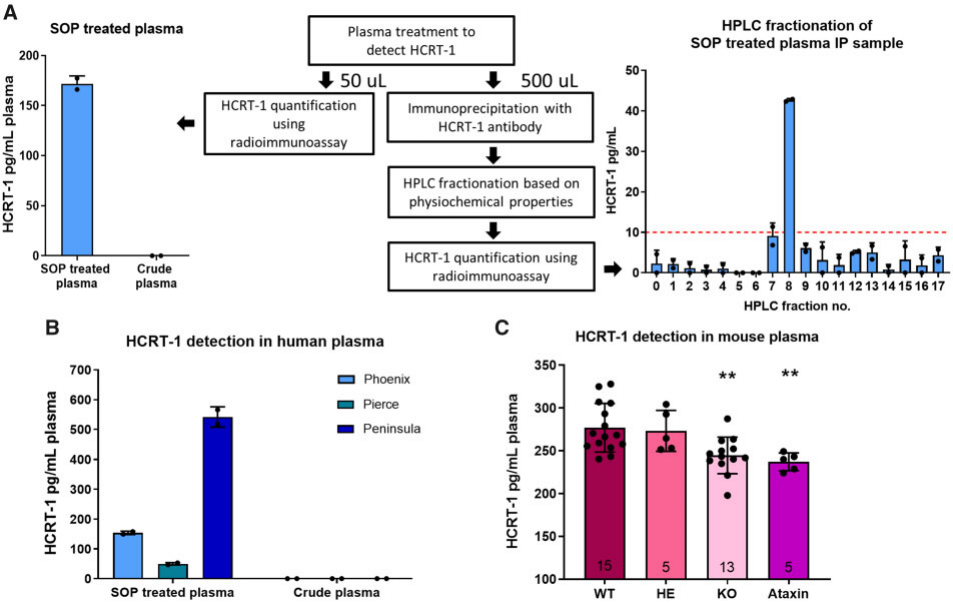
**Verification of signal using different approaches.** (**A**) HPLC fractionation of SOP treated plasma confirms HCRT-1 signal. *Left*: HCRT-1 levels of plasma sample used for IP and HPLC after SOP treatment or no treatment. *Right*: IP of 10 SOP treated plasma samples performed according to RIA protocol followed by HPLC fractionation of pooled IP sample and HCRT-1 quantification of each individual HPLC fraction by RIA. (**C**) Hcrt-1 concentration in plasma of wild-type (WT), heterozygous *Hcrt*-KO (HE), homozygous *Hcrt*-KO (KO) and *Hcrt-Atxn3* (Ataxin) mice following SOP treatment, *n* = 5−15. One-way ANOVA with Dunnett’s multiple comparisons test: overall effect *F*(3,34) = 6.34, *P* = 0.0016. **WT versus KO, *P* = 0.003; WT versus Ataxin, *P* = 0.008. HCRT-1 = hypocretin-1; HPLC = high-performance liquid chromatography; IP = immunoprecipitation; RIA = radioimmunoassay; SOP = standard operation procedure.

To confirm the presence of genuine HCRT-1 in SOP treated plasma samples, we performed IP of SOP treated plasma with the Phoenix antibody from the RIA kit and performed HPLC separation of the sample followed by detection of HCRT-1 in the HPLC fractions (Fig. 3A). As expected from previous HPLC runs (Fig. 1B), HCRT-1 was detectable in fraction 8 (Fig. 3A). It was necessary to pool 10 IP samples to get a detectable signal confirming the very low levels of HCRT-1 in plasma. Figure 3A also shows the HCRT-1 concentration following SOP treatment or no treatment of the same plasma sample used for IP/HPLC.

To further validate the HCRT-1 RIA signal, we tested three different commercially available HCRT-1 antibodies ability to detect plasma HCRT-1 in RIA settings. All three antibodies were able to detect HCRT-1 in plasma following SOP treatment but not in crude plasma (Fig. 3B). However, the concentration of HCRT-1 in SOP treated plasma varied depending on the antibody used. Interestingly, when measuring HCRT-1 in CSF of a healthy control and Narcoleptic Type 1 patient using the three antibodies, markedly different levels of HCRT-1 were found for the control subject (Supplementary Fig. 5A). To further explore the specificity of the antibodies, the Phoenix HCRT-1 peptide in the RIA setting was replaced with a modified HCRT-1 peptide with an extra glutamic acid residue in second position to disrupt N-terminal binding (Supplementary Fig. 6B). A clear difference was observed between standard curves manufactured with the two different peptides (Supplementary Fig. 6C and D). The Phoenix and Pierce antibodies both showed a much lower binding affinity to the modified peptide. The Peninsula antibody was however still able to bind the modified peptide with high affinity. These data confirm that the Phoenix and Pierce antibodies bind to the N-terminal region of HCRT-1, while the Peninsula antibody recognize a different epitope.

### Validation of assay by measurement of Hcrt-1 plasma levels in *Hcrt* knockout mice

To firmly validate the assay, we aimed to investigate if Hcrt-1 plasma levels were decreased in *Hcrt-*KO and *Hcrt-Atxn3* mice compared with WT mice (Fig. 3C). Heterozygous *Hcrt*-KO mice showed similar plasma Hcrt-1 levels compared with WT (Heterozygous: 273.3 ± 23.91 pg/ml plasma, WT mice: 276.9 ± 28.39 pg/ml plasma). Twelve per cent and 14% decreases in plasma Hcrt-1 signals were seen with samples from homozygous *Hcrt*-KO mice and *Hcrt-Atxn3* mice, respectively (Homozygous: 244.6 ± 21.33 pg/ml plasma, *Hcrt-Atxn3*: 237 ± 10.37 pg/ml plasma). For homozygous *Hcrt*-KO compared with WT mice the estimated difference was: 32.28 pg/ml, 95% CI (9.72−54.8), *P* = 0.003. This indicates that the signal: background ration in our assay is around 1:7–8.

We also measured Hcrt-1 in mouse blood using the two other HCRT-1 antibodies mentioned above. With the Pierce antibody, we also saw a significantly decreased signal from *Hcrt*-KO mouse blood (Supplementary Fig. 7), while the Peninsula antibody gave similar signals from WT and *Hcrt-*KO mouse blood. This confirms what we report above, that while the Phoenix and Pierce antibodies show specificity towards the same HCRT-1 epitope, the Peninsula binds to something else in addition. This shows that the Peninsula antibody is not a good antibody for measuring HCRT-1 levels. The Pierce antibody seems to be highly specific and very useful for HCRT-1 detection. Unfortunately, this antibody was discontinued by the vendor limiting the number of experiments we were able to perform with this antibody.

### HCRT-1 levels are similar in blood samples from narcolepsy type 1 patients and controls

**Figure 4:**
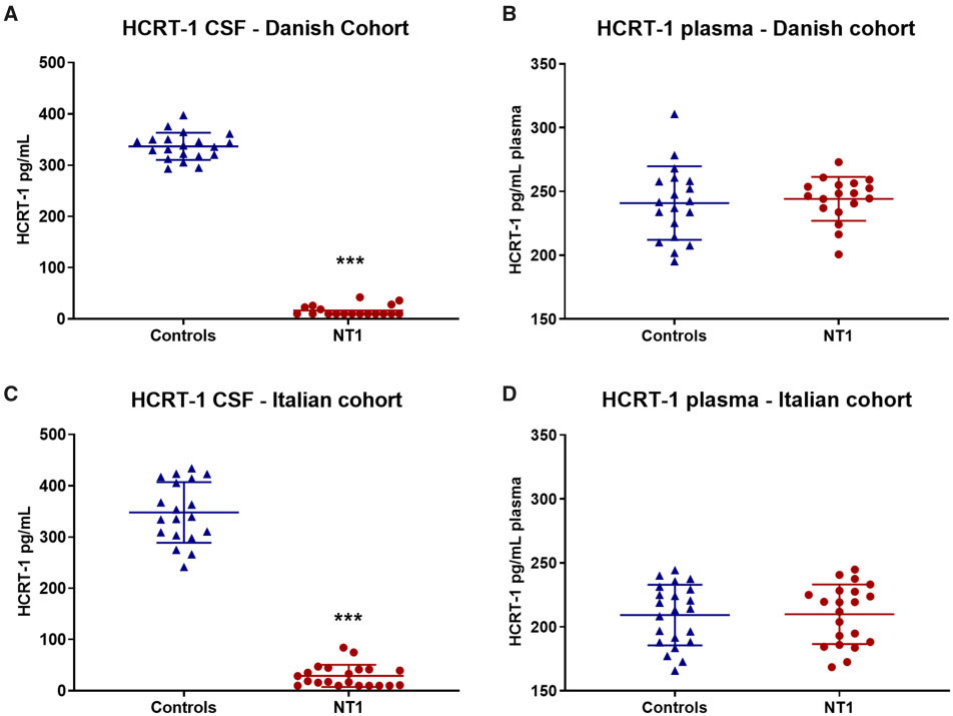
**CSF hypocretin deficiency is not reflected in plasma of Narcoleptic Type 1 patients.** (**A**) CSF and (**B**) plasma HCRT-1 measurements of controls (*n* = 19) and NT1 patients (*n* = 18) from the Danish cohort. ***Student’s *t*-test: *T* = 48.0 df = 36, *P* < 0.001. (**C**) CSF and (**D**) plasma HCRT-1 measurements of controls (*n* = 22) and NT1 patients (*n* = 21) from the Italian cohort. ***Student’s *t*-test: *T* = 23.1 df = 38, *P* < 0.001. Plasma samples were SOP treated prior to quantification by RIA, whereas CSF measurements were analysed without pre-treatment. HCRT-1 = hypocretin-1; NT1 = narcolepsy type 1; RIA = radioimmunoassay; SOP = standard operating procedure.

To explore the feasibility of our assay to be used as a diagnostic tool for identifying NT1 patients, we measured HCRT-1 plasma levels in samples from two independent clinical cohorts. Plasma levels of HCRT-1 in 18 NT1 (Danish cohort) with confirmed low HCRT-1 levels in CSF (Fig. 4A) were similar to the levels detected in a matched control group (NT1: 244.2 ± 17.25 pg/ml plasma and controls: 240.9 ± 28.78 pg/ml plasma, ns) (Fig. 4B). Minimum and maximum values in the cohort were 195.2 and 310.7 pg/ml plasma. To elucidate possible factors affecting plasma HCRT-1 levels, we performed a multiple regression analysis with age, gender, body mass index, diagnosis, albumin concentration and storage time as predictors (Table 2). None of the predictor variables showed a significant *P*-value, suggesting that the strength of the effect on plasma HCRT-1 concentration is low for all variables. Our finding of normal HCRT-1 levels in NT1 patient plasma was replicated in an independent clinical cohort from Bologna, Italy. Again HCRT-1 levels in plasma did not differ between the two groups (NT1: 209.8 ± 23.3 pg/ml plasma and controls: 209.2 ± 23.65 pg/ml plasma, ns) (Fig. 4D). Minimum and maximum values in the Italian cohort were 165.8 and 244.8 pg/ml plasma. For the multiple regression analysis, age, gender, body mass index and diagnosis were tested as predictors. None of the predictors had an effect on HCRT-1 plasma levels in this model (Table 2).

**Table 2:** Predictors of plasma HCRT-1 levels summary of multiple linear regression analyses

Variable	Coefficient estimate	Coefficient std. error	95% CI of estimate	*P*-value
Danish cohort				
Intercept	239.0	30.4	176.9 to 301.1	<0.001
Age (years)	−0.03	0.33	−0.70 to 0.64	0.93
Gender (male)	−8.69	10.0	−29.1 to 11.8	0.39
Body mass index (kg/m^2^)	−0.40	0.99	−2.43 to 1.63	0.69
Diagnosis (NT1)	6.66	9.74	−13.2 to 26.5	0.50
Albumin concentration (g/dL)	4.41	5.81	−7.46 to 16.27	0.45
Italian cohort				
Intercept	195.3	18.2	158.4 to 232.2	<0.001
Age (years)	−0.36	0.29	−0.95 to 0.22	0.22
Gender (male)	−1.03	7.84	−16.9 to 14.8	0.90
Body mass index (kg/m^2^)	0.96	0.87	−0.79 to 2.72	0.27
Diagnosis (NT1)	−1.74	7.49	−16.9 to 13.4	0.82
Italian cohort—NT1 patients only				
Intercept	100.9	44.0	7.69 to 194.2	0.036
Age (years)	−0.94	0.44	−1.87 to 0.057	0.049
Body mass index (kg/m^2^)	3.12	1.53	−0.12 to 6.36	0.058
CSF HCRT-1 concentration (pg/ml)	0.45	0.22	−0.0045 to 0.91	0.052
Time of blood sampling after disease onset (months)	4.32	1.62	0.88 to 7.76	0.017

Multiple linear regression analyses showed no significant effect of any of the predictors in the two cohorts. However, sub-analysis of the NT1 patients in the Italian cohort revealed a significant effect of the time of blood sampling after disease onset and age. This model could significantly predict plasma HCRT-1 level. Level of significance at <0.05 was considered statistically significant.

We also measured HCRT-1 levels in NT1 patients and controls in the Italian cohort by use of a different HCRT-1 antibody (the Pierce antibody) and confirmed our finding of similar HCRT-1 levels between the two groups (Supplementary Fig. 7). In contrast to the Danish cohort, the Italian NT1 blood samples were obtained close to onset of disease. To evaluate the effect of disease length on HCRT-1 plasma levels in these patients, we performed a multiple linear regression analysis where time of blood sampling after disease onset (months) was included as predictor. The analysis showed a significant effect of age and time of blood sampling after disease onset. HCRT-1 increased with time after disease onset but at the same time showed a small decrease with age. The overall *F*-test showed that this model, at a significance level of 0.05, can predict plasma HCRT-1 level [*F*(4,16) = 3.18, *P* = 0.042]. When HCRT-1 levels were measured with the Pierce antibody, there were no significant effects of neither age nor time of blood sampling (Supplementary Tables 1 and 2).

## Discussion

In this study, we have developed a simple reproducible assay to evaluate HCRT-1 levels in blood. We found that heating plasma at pH 8 enabled reproducible detection of HCRT-1. Using HPLC, different antibodies and mice lacking hypocretin, we confirmed the HCRT-1 signal from blood samples, but we also showed that the signal: background ratio in our assay is low—in the order of 1:7. From our measurements on blood samples from mice lacking hypocretin we estimated that HCRT-1 is present in plasma in a concentration of ∼32 pg/ml. Using our assay, we could measure identical signals of HCRT-1 immunoreactivity in plasma samples from NT1 patients and controls in two independent clinical cohorts. Additionally, no correlation was observed between blood and CSF HCRT-1 levels in any of the two groups. The data presented here suggest that peripheral HCRT-1 is intact in NT1 and thus blood HCRT-1 measurements cannot be used as diagnostic tool to identify NT1. It further suggests that the NT1 disease process primarily involve CNS HCRT neurons whereas potential peripheral HCRT producing cells are intact.

We suggest that the increase in signal we see following heating of plasma to 65°C is due to release of HCRT-1 from its blood carriers. Albumin, the most abundant carrier protein in the blood, has indeed been shown to go through a phase shift at this temperature.^28–30^ In addition, it has been shown that a vast majority of the low molecular weight biomarkers detectable in blood are bound to large carrier proteins, representing a sub-proteome itself. Mass spectrometry analysis has shown that removing high molecular weight proteins also removes a substantial amount of the low-molecular weight proteins.^31^^,^^32^ This could explain why so far, no mass spectrometry-based method has been developed for measuring HCRT-1 in blood, as it is a common procedure to remove proteins from plasma, before attempting peptide detection with mass spectrometry.

A limitation of our assay is the high unspecific background signal. This is likely caused by unspecific binding of the antibodies to blood proteins that change antigenicity from denaturation during the heating step. The unspecific signal does not come from albumin, as heating albumin alone in isolated form did not produce a signal. Heating of CSF did produce a slight increase in the signal from our assay, but not at all to the extend we see in blood. We did try different filtering steps to bring this background signal down, but with any filtering the HCRT-1 signal was also lost. This is likely because HCRT-1 is very hydrophobic and sticks to the filter/tubes etc.

We expect that the background noise is in the same order in mice and humans. The estimation of blood Hcrt-1 levels around 30 pg/ml would thus apply to both mice and humans. Since the normal HCRT-1 RIA has a detection level for reliable detection at 40 pg/ml, this explains why HCRT-1 cannot be detected in blood by RIA without pre-treatment.

Our finding of intact peripheral HCRT-1 in NT1 raises numerous questions regarding the origin of peripheral HCRT-1, as well as the aetiology of NT1. Importantly, the data suggest that peripheral HCRT-1 does not solely originate from the CNS and that there instead is a peripheral source of HCRT-1. Intact peripheral HCRT-1 in NT1 would mean that peripheral HCRT-1 producing cells are left unaffected by the autoimmune attack that destroys the HCRT neurons in the brain, the hallmark of NT1 pathology. The implication of this would be that HCRT itself is not the target of the autoimmune attack. Another protein expressed in the HCRT neurons but not the peripheral producers would in this case be the primary target, resulting in a selective destruction of the central HCRT producing neurons. A study of cytotoxic T cells targeting proteins in HCRT neurons did indeed suggest other factors than HCRT-1 to be the autoimmune target,^33^ but other papers have demonstrated direct targeting of HCRT-1.^34–36^ So far, no studies have demonstrated direct *in vivo* targeting of HCRT neurons, so the nature of the primary autoimmune target in NT1 remains unknown.

An alternative explanation to our finding could be that the HCRT-1 producing neurons in hypothalamus are more sensitive and prone to death following the attack, whereas peripheral HCRT-1 producing cells might recover more easily. This could result in low blood HCRT-1 levels at disease onset and then a recovery over time. A low HCRT-1 blood level could in this case be indicative of an active autoimmune process. We did see a correlation between time from disease onset and plasma HCRT-1 levels in our Italian cohort with lower levels closer to onset. This effect was, however, small, and more studies are needed to determine if this is a reproducible effect.

No significant correlations were observed between HCRT-1 plasma concentration and BMI, age, gender, albumin concentration, diagnosis or CSF HCRT-1 concentration in any of the cohorts. These findings contrast with previous studies showing a correlation with a number of these factors (Supplementary Table 1). However, discrepancies among these previous studies are found as well. Adam et al.^37^ report lower blood HCRT-1 levels in obese compared with lean subjects. Baranowska et al.^38^ and Tomasik et al.^39^ report the same findings in women and children during puberty, respectively. In contrast, Heinonen et al.^40^ observed significantly higher HCRT-1 levels in obese subjects. These discrepancies may be explained by the different assays used, the sensitivity of the assays and potentially the use of different antibodies. In the present study, we observed significantly different HCRT-1 levels in both blood and CSF when measuring the same sample with different antibodies. Using Phoenix and Pierce antibody we found that the HCRT-1 levels in blood of the controls were lower than CSF HCRT-1 levels, which is in contrast to what we observed with the Peninsula antibody. With the Peninsula antibody we observed surprisingly high HCRT-1 blood levels, while measuring CSF HCRT-1 levels with the Peninsula antibody showed lower CSF HCRT-1 levels in comparison. We speculate that these differences might arise from the Peninsula antibody having a strong cross-reactivity with other components of plasma. The difference in signal could also be due to modified or degraded variants of HCRT-1 in blood or CSF. It has previously been shown that fractions of HCRT-1 are present in CSF,^41^ but whether this is true for plasma is not known. Importantly, our data demonstrate that one should take caution when comparing HCRT-1 levels detected with different antibodies/assays.

Despite the lack of a relationship between disease state and HCRT-1 plasma levels in NT1, our assay provides a solid platform for future studies on the peripheral actions of HCRT-1. Measuring HCRT-1 levels in blood could be relevant in a range of diseases besides NT1 and might help determine the role of peripheral HCRT-1. The narrow concentration range of HCRT-1 in blood could suggest a tight regulation, supporting the idea of HCRT-1 as a peptide hormone. Where peripheral HCRT is produced and how this is regulated is still unknown and needs further attention.

## Supplementary material

Supplementary material is available at *Brain Communications* online.

## Funding

The project was funded by an investigator driven grant from UCB Pharma and by Proof of Concept (PoC) funding from Region Hovedstadens Tech Transfer Office.

## Competing interests

The authors report no competing interests.

## Supplementary Material

fcab050_Supplementary_Data

## Abbreviations

BSA =: bovine serum albumin
EDTA =: ethylenediaminetetraacetic acid
HCRT =: hypocretin
Hcrt-1/HCRT-1 =: hypocretin-1
HCRT-2 =: hypocretin-2
HPLC =: high-performance liquid chromatography
IP =: immunoprecipitation
NT1 =: narcolepsy type 1
RIA =: radioimmunoassay
SOP =: standard operating procedure
SPE =: solid phase extraction
WT =: wild-type
