# Supplementary material for: Pre-treatment of blood samples reveal normal blood hypocretin/orexin signal in narcolepsy type 1
Source: Brain Commun. 2021 Mar 22;3(2):fcab050. doi: 10.1093/braincomms/fcab050 (PMC8100001; doi:10.1093/braincomms/fcab050)
Supplement: fcab050_Supplementary_Data [file fcab050_supplementary_data.pdf]

Supplementary figures and tables.

Manuscript: Pre-treatment of blood samples reveal normal blood hypocretin/orexin signal in Narcolepsy Type 1.

Short title: Intact peripheral HCRT-1 in Narcolepsy Type 1

Supplementary tables: 4

Supplementary figures: 7

Helene M. Ægidius,[1] Lars Kruse,[2] Gitte L. Christensen,[2] Marc P. Lorentzen,[2] Niklas R. Jørgensen,[2] Monica Moresco,[3] Fabio Pizza,[3,4] Giuseppe Plazzi,[3,5,] Poul J. Jennum,[6] Birgitte R. Kornum[1]

1 Department of Neuroscience, University of Copenhagen, Denmark

2 Department of Clinical Biochemistry, Rigshospitalet, Glostrup, Denmark

3 IRCCS Istituto delle Scienze Neurologiche di Bologna, Bologna, Italy

4 Department of Biomedical and Neuromotor Sciences (DIBINEM), University of Bologna, Bologna, Italy

5 Department of Biomedical, Metabolic and Neural Sciences, University of Modena and Reggio-Emilia, Modena, Italy.

6 Danish Center for Sleep Medicine, Department of Clinical Neurophysiology, Rigshospitalet, Glostrup, Denmark

Supplementary table 1: Plasma HCRT-1 concentrations measured in human subjects.

Group	Plasma pretreatment	Assay used for HCRT-1 quantification	HCRT-1 concentration (mean ± SD/SEM)	Detection limit	Conclusion	Reference
Narcolepsy and healthy	SPE	RIA Phoenix pharmaceuticals	175 to 847 pg/ml (reported in article as a range)	40.0 pg/ml	Normal plasma levels in Narcolepsy	Dalal et al.[1]
Narcolepsy and healthy	SPE	RIA Peninsula Lab (RIK9600)	20.83 ± 4.34 pg/ml 26.67 ± 3.23 pg/ml	Reliable measurement down to 10.0 pg/ml	Plasma HCRT-1 is lower in people with narcolepsy	Higuchi et al.[2]
Healthy	Acidification, SPE	RIA	1.94 ± 0.24 pmol/l	Assay could detect changes of 0.97 ± 0.21 fmol/tube from zero	-	Arihara et al.[3]
Healthy	Not reported	RIA Peninsula lab (RIK9600)	29.9 ± 1.6 pg/ml	Assay sensitivity 18.0 pg/ml	HCRT-1 increases following fasting	Komaki et al.[4]
Healthy	Not reported	RIA Peninsula Lab	3.4 to 16.6 pmol/l	Lowest detectable concentration was 2.8 pmol/l	Plasma HCRT-1 increases with age	Matsumura et al.[5]
Healthy (men)	SPE	EIA (ELISA) Phoenix pharmaceuticals	2610±187 pg/ml (basal value)	Lowest detectable concentration was 370 pg/mL	Plasma HCRT-1 is increased during exercise	Messina et al.[6]
Healthy (women 28-32 and 48- to 57-years old)	Not reported	RIA Peninsula Lab	2438100 ± 688800 pg/ml 7056100 ± 1656200 pg/ml	Not reported	Higher plasma HCRT-1 levels are associated with hypoestrogenism. Plasma HCRT-1 levels directly correlate with BMI	El-Sedeek et al.[7]
Healthy (children during puberty)	Acidification, SPE	EIA Peninsula Lab	1010 ± 120 pg/ml	Assay sensitivity was 60 pg/ml	Plasma HCRT-1 negatively correlates with BMI and positively correlates with caloric demand	Tomasik et al.[8]
Obese children	Acidification, SPE	RIA	33.3 ± 1.97 pg/ml	Lowest detectable	Plasma HCRT-1 increases after 5-	Bronsky et al.[9]

		Phoenix pharmaceuticals		concentration was 4 pg/ml	week weight loss program. Negative correlation between HCRT-1 and age, height, body weight and BMI	
Lean and Obese	Acidification, SPE	RIA<br>Phoenix pharmaceuticals	40-61.4 pg/ml (reported in article as a range)	Lowest detectable concentration was 40 pg/ml	Plasma HCRT-1 level is decreased in obese individuals	Adam et al.[10]
Lean and Obese	SPE	EIA (ELISA)<br>Phoenix pharmaceuticals	0.8 ± 0.4 pg/ml<br>75.3 ± 24.1 pg/ml	The lowest detectable concentration was 370 pg/ml	Higher plasma HCRT-1 levels in obese, Plasma HCRT-1 positively correlates with BMI	Heinonen et al.[11]
Healthy and OSAHS	Not reported	RIA<br>Peninsula Lab	32.3 ± 1.3 pg/ml<br>36.3 ± 1.2 pg/ml	Reliable measurement down to 10.0 pg/ml	Plasma HCRT-1 is higher in people with OSAHS. Plasma HCRT-1 does not correlate with age and BMI	Igarashi et al.[12]
Healthy and OSAHS	SPE	RIA	12.3 ± 1.9 pmol/l<br>4.9 ± 0.8 pmol/l	Assay could detect changes of 0.97 ± 0.21 fmol/tube from zero	Plasma HCRT-1 is lower in people with OSAHS	Sakurai et al.[13]
Healthy and OSAS	SPE	RIA<br>Phoenix Pharmaceuticals	20.6 ± 4.5 pg/ml<br>9.4 ± 1.9 pg/ml	Not reported	Plasma HCRT-1 is lower in people with OSAS	Busquets et al.[14]
Healthy and OSA	None	EIA<br>Phoenix Pharmaceuticals	3140 ± 650 pg/ml<br>1500 ± 460 pg/ml	Assay sensitivity was 370 pg/ml	Plasma HCRT-1 is lower in people with OSA	Aksu et al.[15]
Healthy and AN-R	Acidification, SPE	RIA<br>Phoenix pharmaceuticals	~70 pg/ml (mean)<br>~40 pg/ml (mean)<br>(Group means not reported in article)	Lowest detectable level was 1 pg/ml	Plasma HCRT-1 is lower in AN-R subjects	Janas-Kozik et al.[16]

Healthy and schizophrenic	Acidification, SPE	RIA Phoenix pharmaceuticals	38.8 ± 15.5 pg/ml 60.7 ± 37.9 pg/ml	Not reported	Plasma HCRT-1 is higher in people with schizophrenia	Ling-Chien et al.[17]
Healthy and PTSD	Acidification, SPE	EIA (ELISA) Peninsula Lab	2500 ± 500 pg/ml 1300 ± 500 pg/ml	Not reported	Plasma HCRT-1 is lower in PTSD veterans	Strawn et al.[18]

Abbreviations: AN-R: anorexia nervosa; EIA: Enzyme immunoassay; OSA(S): Obstructive sleep apnea syndrome; OSAHS: Obstructive sleep apnea- Hypopnea syndrome; PSTD: Post traumatic stress disorder; RIA: Radioimmunoassay; SD: Standard deviation; SEM; Standard error of the mean; SPE: Solid phase extraction

References for supplementary table 1:

1. Dalal MA, Schuld A, Haack M, et al. Normal plasma levels of orexin A (Hypocretin-1) in narcoleptic patients. *Neurology*. 2001;56:1749-51.
2. Higuchi S, Usui a, Murasaki M, et al. Plasma orexin-A is lower in patients with narcolepsy. *Neurosci Lett*. 2002;318(2):61-4.
3. Arihara Z, Takahashi K, Murakami O, et al. Immunoreactive orexin-A in human plasma. *Peptides*. 2001;22(1):139-42.
4. Komaki G, Matsumoto Y, Nishikata H, et al. Orexin-A and leptin change inversely in fasting non-obese subjects. *Eur J Endocrinol*. 2001;144(6):645-651.
5. Matsumura T, Nakayama M, Nomura A, et al. Age-related changes in plasma orexin-A concentrations. *Exp Gerontol*. 2002;37(8-9):1127-1130.
6. Messina G, Di Bernardo G, Viggiano A, et al. Exercise increases the level of plasma orexin A in humans. *J Basic Clin Physiol Pharmacol*. 2016;27(6):611-616.
7. El-Sedeek M, Korish AA, Deef MM. Plasma orexin-A levels in postmenopausal women: Possible interaction with estrogen and correlation with cardiovascular risk status. *BJOG An Int J Obstet Gynaecol*. 2010;117(4):488-492.
8. Tomasik PJ, Spodaryk M, Sztefko K. Plasma concentrations of orexins in children. *Ann Nutr Metab*. 2004;48(4):215-220.
9. Bronský J, Nedvídková J, Zamrazilová H, et al. Dynamic changes of orexin A and leptin in obese children during body weight reduction. *Physiol Res*. 2007;56(1):89-96.
10. Adam J, Menheere P, van Dielen F, Soeters P, Buurman W, Greve J. Decreased plasma orexin-A levels in obese individuals. *Int J Obes*. 2002;26(2):274-276.
11. Heinonen M V., Purhonen AK, Miettinen P, et al. Apelin, orexin-A and leptin plasma levels in morbid obesity and effect of gastric banding. *Regul Pept*. 2005;130(1-2):7-13.
12. Igarashi N, Tatsumi K, Nakamura A, et al. Plasma Orexin-A Levels in Obstructive Sleep Apnea-Hypopnea Syndrome. *Chest*. 2003;124(4):1381-1385.

13. Sakurai S, Nishijima T, Takahashi S, Yamauchi K, Arihara Z, Takahashi K. Clinical significance of daytime plasma orexin-A-like immunoreactivity concentrations in patients with obstructive sleep apnea hypopnea syndrome. *Respiration*. 2004;71(4):380-384.

14. Busquets X, Barbé F, Barceló A, et al. Decreased plasma levels of orexin-A in sleep apnea. *Respiration*. 2004;71(6):575-579.

15. Aksu K, Güven SF, Aksu F, et al. Obstructive sleep apnoea, cigarette smoking and plasma orexin-A in a sleep clinic cohort. *J Int Med Res*. 2009;37(2):331-340.

16. Janas-Kozik M, Stachowicz M, Krupka-Matuszczyk I, et al. Plasma levels of leptin and orexin A in the restrictive type of anorexia nervosa. *Regul Pept*. 2011;168(1-3):5-9.

17. Chien YL, Liu CM, Shan JC, et al. Elevated plasma orexin A levels in a subgroup of patients with schizophrenia associated with fewer negative and disorganized symptoms. *Psychoneuroendocrinology*. 2015;53:1-9.

18. Strawn JR, Pyne-Geithman GJ, Ekhator NN, et al. Low cerebrospinal fluid and plasma orexin-A (Hypocretin-1) concentrations in combat-related posttraumatic stress disorder. *Psychoneuroendocrinology*. 2010;35(7):1001-1007.

Supplementary table 2: High-performance liquid chromatography program.

**System**	Shimadzu instrument
**Column**	Waters Xselect CSH C18, 130Å, 3.5 µm, 4.6 mm X 100 mm
**Mobile phase A**	95% Water, 5% MeCN (0.1% TFA)
**Mobile phase B**	100% MeCN (0.1% TFA)
**Linear gradient**	10%-100% (0-27 min)
**Fraction collection start**	2 min (fraction 0)
**Fraction collection stop**	20 min (fraction 17)
**Fraction evaporation**	N_2 evaporation at 37°C for 3 hours
**Flow rate**	1 mL/min
**Column temperature**	30 C°

Supplementary tables 3 + 4: Effects of different variables on plasma HCRT-1 levels when measured using the Pierce antibody.

Italian cohort				
**Variable**	**Coefficient estimate**	**Coefficient std. error**	**95% CI of estimate**	**p-value**
Intercept	77.2	15.1	46.6 to 107.8	<0.001
Age (years)	-0.050	0.24	-0.54 to 0.44	0.84
Gender (male)	9.91	6.50	-3.25 to 23.1	0.14
Body mass index (kg/m^2)	-0.33	0.72	-1.78 to 1.12	0.65
Diagnosis (NT1)	0.52	6.21	-12.1 to 13.1	0.93

Italian cohort – NT1 patients only				
**Variable**	**Coefficient estimate**	**Coefficient std. error**	**95% CI of estimate**	**p-value**
Intercept	41.3	48.8	-62.1 to 144.68	0.41
Age (years)	-0.51	0.49	-1.55 to 0.52	0.31
Body mass index (kg/m^2)	0.74	1.70	-2.85 to 4.33	0.67
CSF HCRT-1 concentration (pg/mL)	0.30	0.24	-0.21 to 0.80	0.23
Time of blood sampling after disease onset (months)	2.02	1.80	-1.79 to 5.83	0.28

Supplementary figure 1: High-performance liquid chromatography fractionation of blood samples.

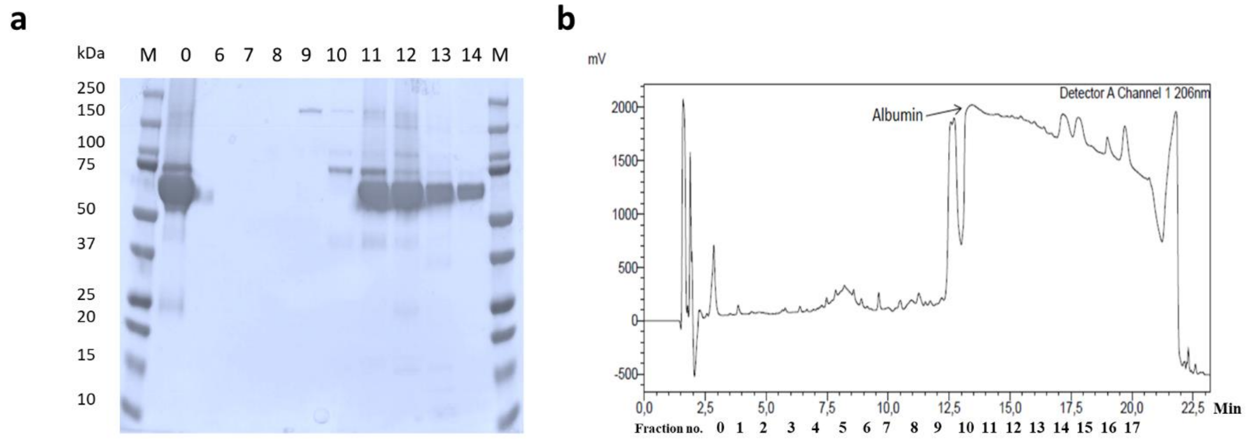

a) HPLC fractionation of 99 ul serum/H_2O (1:1) followed by SDS-page and Coomassie Blue staining of the individual HPLC fractions. Samples were heated for 10 min at 70°C in DDT-containing SDS loading buffer, run on SDS Precast Gel 4-20% and stained by Coomassie blue. **b)** Chromatogram from HPLC run of 99 ul serum/H_20 sample. Fraction number is paired with the time at which sampling of a given fraction ceased. E.g. from minute 13 to 14 fraction 11 was sampled. HPLC = High performance liquid chromatography.

Supplementary figure 2: Non-cropped western blots.

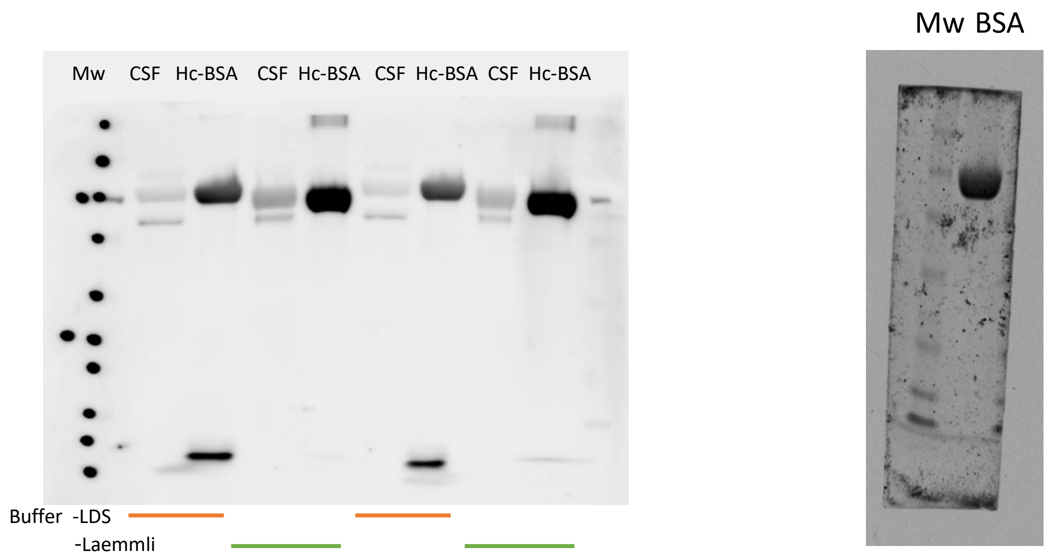

Mw | 1 | 2 | 3 | 4 | 5 | 6 | 7 | 8 | Mw | 20 µg BSA | Mw

1: Human CSF in LDS buffer – heated at 70°C.

2: Hypocretin-1 standard peptide in LDS buffer – heated at 70°C.

3: Human CSF in Laemmli buffer – heated at 70°C.

4: Hypocretin-1 standard peptide in Laemmli buffer – heated at 70°C.

5: Human CSF in LDS buffer – heated at 95°C.

6: Hypocretin-1 standard peptide in LDS buffer – heated at 95°C.

7: Human CSF in Laemmli buffer – heated at 95°C.

8: Hypocretin-1 standard peptide in Laemmli buffer – heated at 95°C.

Supplementary figure 3: Sample pretreatment of HCRT-1 standard.

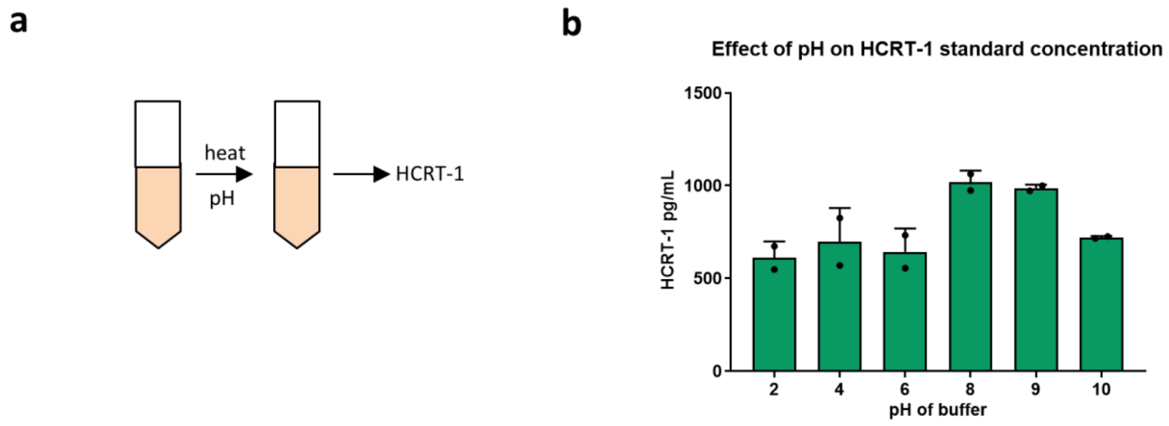

a) Hypothesis: Altering assay conditions affects HCRT-1 binding to carrier proteins. Heat and pH-change facilitate dissociation of HCRT-1 from protein carriers such as BSA in the standard solution. **b)** Effect of heat and pH-change on HCRT-1 standard peptide. Samples were heated for 10 min at 65°C. n = 2. HCRT-1 was quantified by RIA for all samples. BSA = bovine serum albumin; HCRT-1 = Hypocretin-1; RIA = radioimmunoassay.

Supplementary figure 4: CSF HCRT-1 levels of Narcolepsy Type 1 patients and controls without and with standard operating procedure treatment.

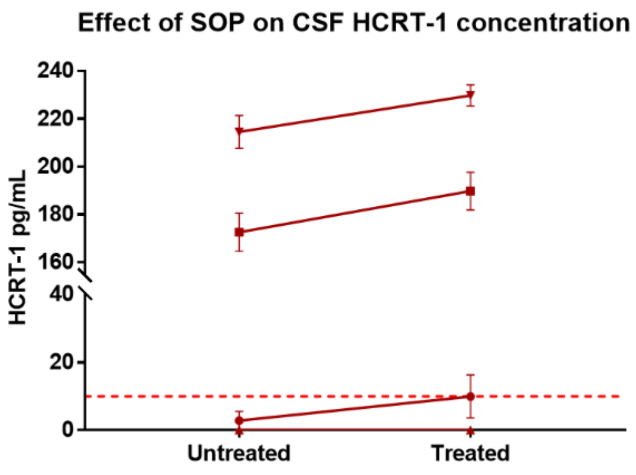

HCRT-1 concentration in CSF samples from NT1 patients (n = 2) and controls (n = 2) before and after SOP treatment. Each set of two dots connected by a line represents 1 individual with standard deviations from the technical duplicates. One of the patients had 0 HCRT-1 at both measurements, so the line is on the x-axis. HCRT-1 = Hypocretin-1; NT1 = Narcolepsy Type 1, SOP = standard operating procedure.

Supplementary figure 5: Assay performance.

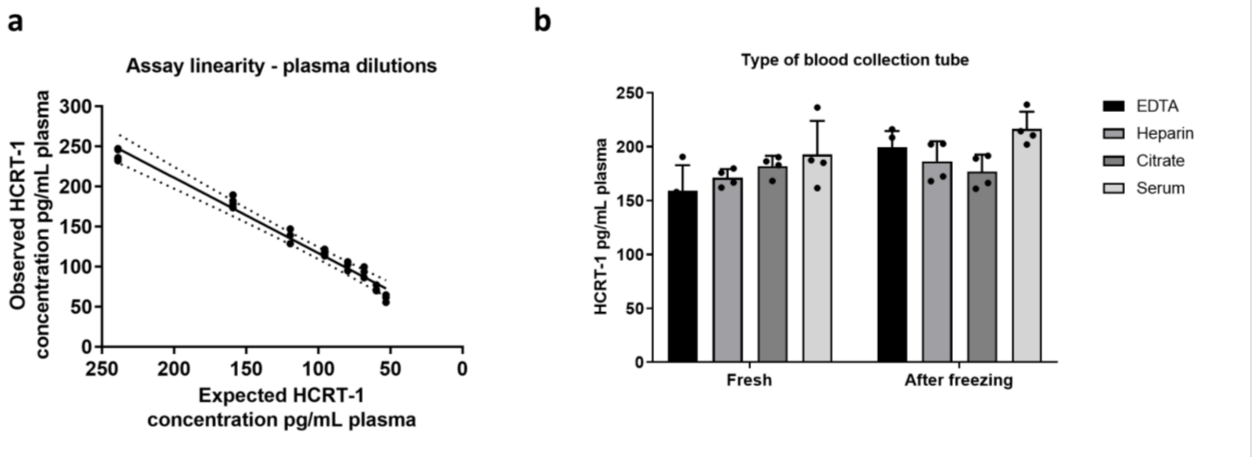

a) Linearity-of-dilution experiment to evaluate how accurately the developed assay measures HCRT-1 in plasma. Correlation between the observed and expected HCRT-1 concentration of 7 different plasma dilutions. Pearson correlation coefficient: 0.991. **b)** The effect of the type of blood collection tubes on detectable plasma or serum HCRT-1 levels. Plasma and serum from two individuals were SOP treated and HCRT-1 levels were quantified. Three plasma anticoagulants were tested: citrate, EDTA and heparin. None of the anticoagulants nor serum changed the detected HCRT-1 levels. n = 4. Samples were either analyzed fresh or after freezing (-20°C). Shown is mean + SD. HCRT-1 = Hypocretin-1; SOP = standard operating procedure.

Supplementary figure 6: HCRT-1 signal specificity in CSF using three different antibodies.

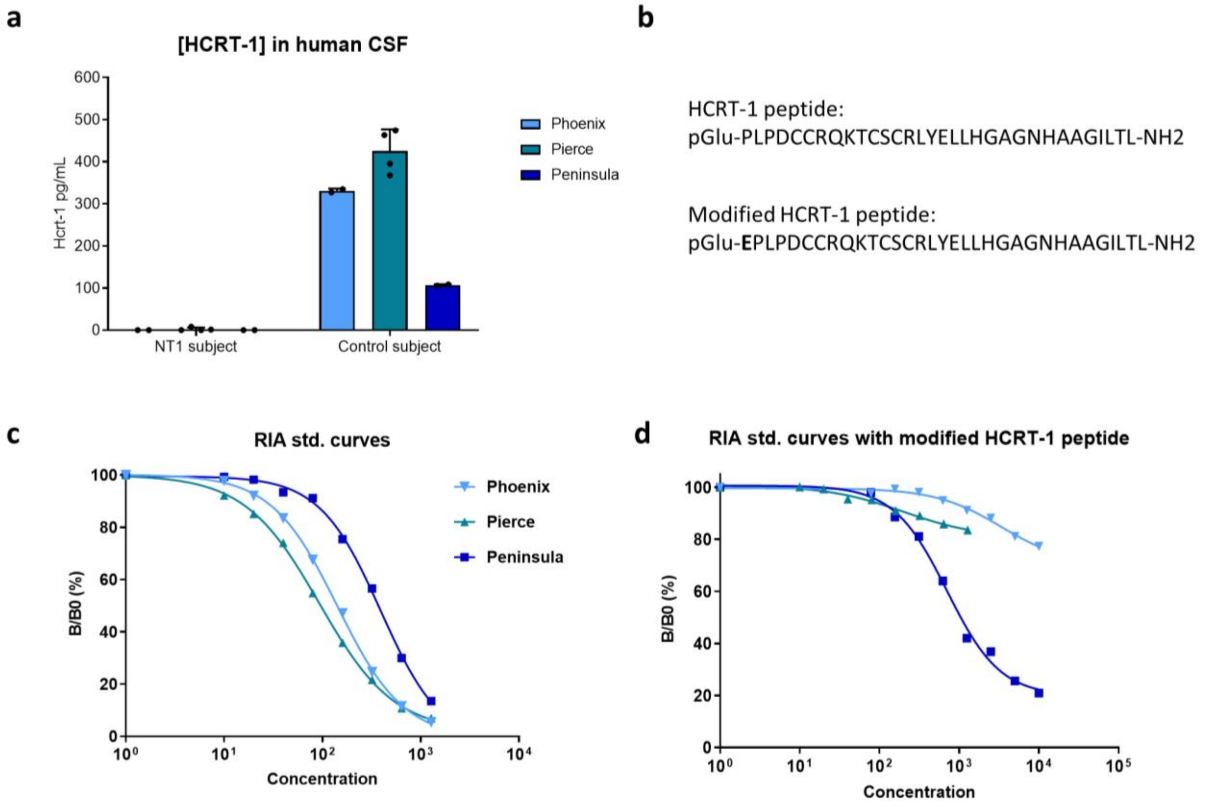

a) CSF HCRT-1 concentration as quantified by RIA using Phoenix, Pierce, and Peninsula antibodies. Two patient samples were quantified with each antibody. Shown is mean + SD. **b)** Modification of the amino acid sequence of HCRT-1 peptide. **c)** RIA standard curves manufactured with kit HCRT-1 standard (Phoenix) and Phoenix, Pierce, and Peninsula antibodies. **d)** RIA standard curves manufactured with modified HCRT-1 standard and Phoenix, Pierce, and Peninsula antibodies. Shown in c and d are means of technical duplicate measurements. HCRT-1 = Hypocretin-1; RIA = radioimmunoassay.

Supplementary figure 7: Hcrt-1 /HCRT-1 immunoreactivity in plasma from *Hcrt* knockout and wild type mice and Narcolepsy Type 1 patients and controls with alternative antibodies.

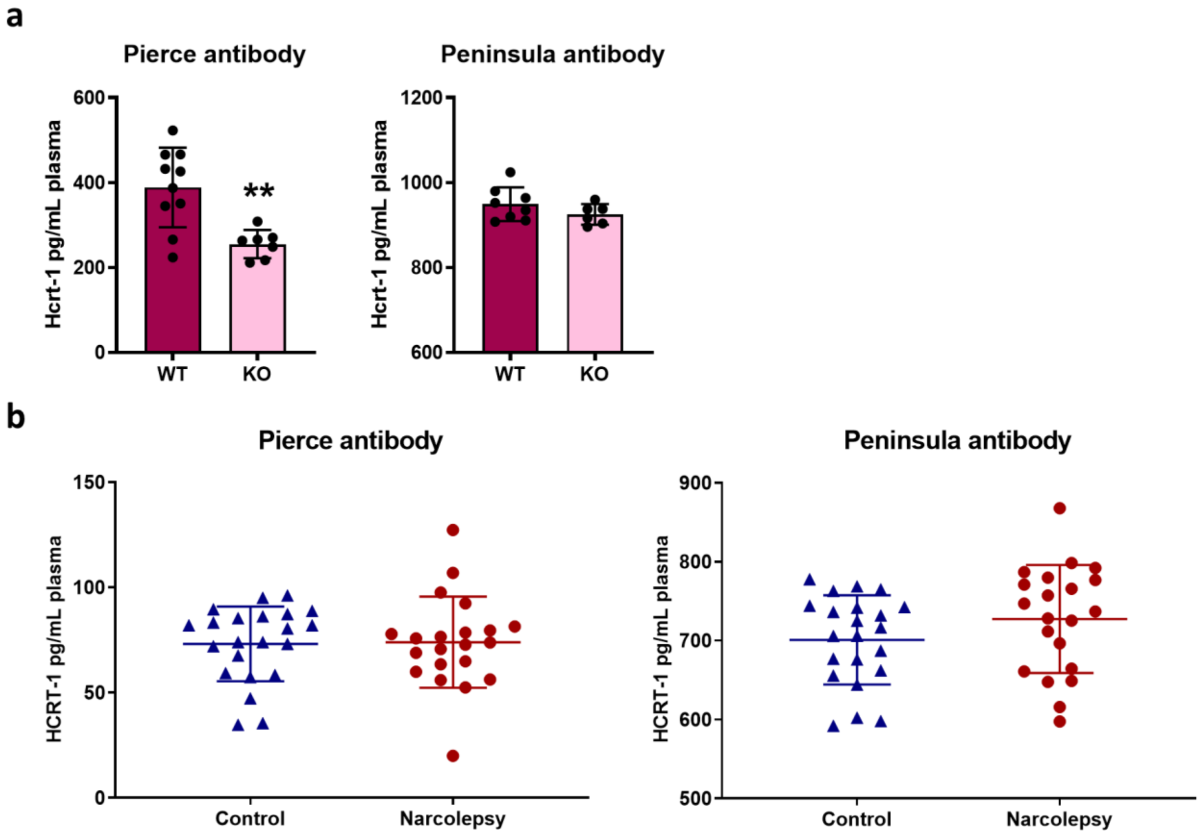

a) Plasma Hcrt-1 concentration of mouse samples as quantified by RIA using antibodies from Pierce (#PA124892, Thermo Scientific – discontinued), and Peninsula (#T-4072.0500, Peninsula Laboratories International). WT: wild type mice, KO: Homozygous *Hcrt* knockout mice. Shown is mean + SD. ** Two tailed unpaired t test with Welch's correction t=4.15, df=11.96, p=0.0014.
b) Plasma HCRT-1 concentration of human samples from the Italian cohort as quantified by RIA using Pierce and Peninsula antibodies. Shown is mean + SD. Hcrt-1/HCRT-1 = Hypocretin-1; RIA = radioimmunoassay.
